# Adherence to the Mediterranean Diet Association with Serum Levels of Nitric Oxide, Prostacyclin, and Thromboxane B_2_ among Prinzmetal Angina Patients and Healthy Persons

**DOI:** 10.3390/nu15030738

**Published:** 2023-02-01

**Authors:** Mahsa Mohajeri, Arrigo F. G. Cicero

**Affiliations:** 1 Digestive Disease Research Center, Ardabil University of Medical Sciences, Ardabil 56131-56491, Iran; 2 Medicine and Surgery Sciences Department, Alma Mater Studiorum University of Bologna, 40123 Bologna, Italy; 3 IRCCS AOU S. Orsola-Malpighi University Hospital, 40123 Bologna, Italy

**Keywords:** Prinzmetal angina, Nitric oxide, Prostacyclin, Thromboxane B_2_, Mediterranean diet

## Abstract

This study aimed to assess the association between adherence to the Mediterranean diet with serum Nitric oxide, Prostacyclin, and Thromboxane B_2_ among Prinzmetal angina patients and healthy persons. This case-control study was conducted among 100 Prinzmetal angina patients and 100 healthy persons referred to the Ardabil Imam Khomeini hospital between 2021 and 2022. Blood samples were obtained from all study participants for measurement of serum Nitric oxide, Prostacyclin, and Thromboxane B_2_. To calculate adherence to the Mediterranean diet, the ten-item screener was used. The serum Nitric oxide in patients who adhered more to the Mediterranean diet was higher than patients with less adherence (coeff. = 0.41 *p* = 0.04). The serum Prostacyclin level in patients with greater adherence to the Mediterranean diet was 0.34 units higher than patients with less adherence (coeff. = 0.34 *p* = 0.02). The level of serum Thromboxane B_2_ had a negative association with adherence to the Mediterranean diet (coeff. = −0.48 *p* = 0.04). The amount of consumption of olive oil, fruits, vegetables, and legumes in healthy people was more than Prinzmetal angina patients. In Prinzmetal angina patients, more adherence to the Mediterranean diet can decrease the serum Thromboxane B_2_ and increase the serum Nitric oxide and Prostacyclin.

## 1. Introduction

In Iran, chronic non-communicable diseases, especially cardiovascular diseases, account for a major portion of the causes of death and disability [1,2]. Deaths caused by cardiovascular diseases in developed countries are decreasing in recent years due to preventive measures and effective interventions [3,4], while these cases are high in developing countries [5]. Economic and industrial development and the expansion of communication have caused mechanization; this trend changes the way of life and increases the incidence of cardiovascular diseases (e.g., coronary heart disease). These changes include smoking, inactivity, and an unhealthy diet [4]. On the other hand, life expectancy is increasing rapidly due to the reduction of communicable diseases in developing countries, and people are exposed to these risk factors for a longer period [6].

Angina is a feeling of discomfort or pain in the chest that lasts for several minutes [7]. This condition occurs when the heart is unable to receive oxygen from the coronary arteries for a short period due to the narrowing of the coronary arteries [8]. Due to the lack of blood reaching the heart muscle, chest pain may be felt only in the morning or sometimes in cold weather, after eating a heavy food meal, after physical activity, and after emotional stress [7,9]. Angina is usually felt in the middle of the chest, but it can also be felt in the shoulders, back, lower jaw, neck, arms, and left hand [10]. There are different types of angina, including constant, non-constant, and Prinzmetal angina [11].

Prinzmetal angina (synonyms: vasospastic angina (VS), variant angina, spontaneous angina) is a rare type of angina pectoris caused by spasms of blood vessels supplying the heart and accompanied by an elevation of the ST segment in the electrocardiogram [12]. The clinical manifestation of spasms of the coronary arteries is the sudden onset of severe pain at rest or during sleep in the morning, less often in the daytime, with no obvious connection with physical stress, often developing at the same time of day [13,14]. The clinical features of this angina are unbearable pain, excessive sweating, tachycardia, and hypotension. Any attack of VS can turn into acute myocardial infarction [15].

One of the causes of Prinzmetal angina syndrome incidence can be the reduction of Nitric oxide production [16,17,18]. Nitric oxide (NO) is produced from L-arginine by the Nitric oxide synthase (NOS) [19]. The constitutive in the arterial endothelium continuously generates NO, which maintains basal vascular tone [20]. Nitric oxide production-related gene mutation can be one of the prinzmetal angina syndrome incidence risk factors [18]. Prostaglandin I_2_ is a vasodilator [21,22] and Thromboxane B_2_ is a vasoconstrictor [23]. Many related studies indicated that the high serum level of Thromboxane B_2_ and low serum of Prostaglandin I_2_ can be some of the main reasons for the occurrence of Prinzmetal angina syndrome [24,25].

The Mediterranean diet’s cardio-protective properties have been linked to increased lifespan and decreased cardiovascular disease incidence and death in southern Europe as compared to northern Europe in the Seven Countries Study [26,27] and the MONICA study [28]. A Mediterranean diet score was created to aid the assessment of the health advantages of this food pattern [29]. The Mediterranean diet involves consuming a lot of olive oil and plant-based foods like fruit, vegetables, legumes, whole-grain cereals, nuts, and seeds. It also includes varying amounts of fish, moderate to high amounts of alcohol (red wine in particular), dairy products, and relatively little meat [especially red meat [27]. Evidence for the preventive impact of the Mediterranean diet on cardiovascular illnesses is growing in observational and intervention studies [30,31]. Intervention studies have shown that the Mediterranean diet decreases risk factors of cardiovascular disease and death in people who have previous myocardial infarctions [32]. According to one study, a 2-point rise in the Mediterranean diet score was strongly associated with a 9% lower risk of cardiovascular disease death [33]. Additionally, the prospective Nurses’ Health Study has demonstrated that the Mediterranean diet reduced the incidence of cardiovascular diseases and stroke [34].

Many studies reported the effect of the Mediterranean diet in heart disease prevention [35,36], but so far, no study has investigated the association between this dietary pattern with the serum level of Prostacyclin, Nitric oxide, and Thromboxane B_2_ in Prinzmetal angina patients. Due to the lack of studies in this field and the importance of identifying effective dietary factors in the prevention and control of Prinzmetal angina, this study assessed and compared the levels of serum Prostacyclin, Nitric oxide, and Thromboxane B_2_ and their association with adherence to the Mediterranean diet among Prinzmetal angina patients and healthy people in Ardabil adults.

## 2. Materials and Methods

This case-control study was conducted among 100 Prinzmetal angina patients aged 40–70 years that were referred to the heart clinic of Ardabil Imam Khomeini hospital between 2021 and 2022. The subjects of the control group (*n* = 100) were matched with the subjects of the patient group based on age, gender, and body mass index (BMI) and were selected from the patients of the skin clinic of Imam Khomeini Hospital in Ardabil.

Patients with congenital heart abnormalities and vascular heart disease, people who are unable to move due to cerebrovascular accidents, patients with chronic inflammatory diseases such as tuberculosis-hepatitis, pregnant women, people treated with birth control pills/steroid replacement, people with kidney failure, patients with thyroid and parathyroid disorders, and people who had any surgery in the last 6 months were excluded from the study. Patients that were referred to the hospital for the first time to diagnose the disease were included in this study and their blood was taken at the beginning of the diagnosis, so that they had not yet received any intervention or medication.

The general characteristics of the study participants (age, weight, height, BMI, gender, marital status, level of education, history of chronic diseases, alcohol and smoking consumption, and physical activity level) were collected by the researcher using a general questionnaire in the form of an interview. Weight, height, and hip and waist circumferences were measured using the standard methods. The hip circumference was measured as the greatest circumference around the buttocks and the waist circumference was measured halfway between the costal edge and the iliac crest. The waist-hip circumference ratio was calculated by dividing the waist circumference by the hip circumference. Body mass index was calculated as weight (kg) divided by squared height (m^2^). Systolic and diastolic blood pressures were measured twice after resting for 15 min in a sitting position [37]. The level of physical activity was assessed using the International Physical Activity Questionnaire (IPAQ) [38].

### 2.1. Physical Activity

According to the IPAQ-S scoring protocol, METs min·wk^−1^ of a specific activity (vigorous, moderate, and low-intensity PA) is computed by multiplying the MET value of particular activity (3.3 for walking, 4.0 for moderate PA, and 8.0 for vigorous PA) with hours spent in that particular activity (walking METs min·wk^−1^ = 3.3 × walking minutes × walking days). Only the activities lasting at least 10 min were considered physical activity. The PA was categorized using the IPAQ scoring protocol. The cut-off levels are based on the current guidelines for PA in the IPAQ scoring protocol as three groups: 1. “low” (some activity is reported but not enough to meet categories 2 or 3), 2. “moderate” (5 or more days of any combination of walking, moderate-intensity or vigorous intensity activities achieving a minimum of at least 600 METs min·wk^−1^) and 3. “high” (7 or more days of any combination of walking, moderate- or vigorous-intensity activities accumulating at least 3000 METs min·wk^−1^).

### 2.2. Nitric Oxide Measurement

Blood samples were obtained from all study participants after fasting for at least 10 h. All the samples were centrifuged at 3000× *g* for 10 min and serum samples were divided into aliquots and stored at −70 °C until analysis. Nitric oxide was measured by the Griess reaction using the micro-pitting method. To measure total Nitrite and Nitrate (NOx) concentrations, 100 microliters of serum were poured into a micro pellet and the samples were kept overnight at 4 °C. At this stage, to separate the protein sediment, the samples were centrifuged for 10 min in 1000× *g* and 100 microliters of the upper phase were kept for measurement. Then, 100 microliters of vanadium chloride solution were kept from the upper phase for NOx measurement. Then, 100 microliters of vanadium chloride solution (8 mg/mL) were added to convert Nitrates into Nitrites. One hundred microliters of each serum were poured into a 96-well plate. One hundred microliters of sulfanilamide solution (1 g in 100 cc of 5% phosphoric acid) were added to all the wells containing the sample and standard. The plate was incubated for 5–10 min in the dark at room temperature. One hundred microliters of N-1 naphthyl ethylene diamine dihydrochloride solution was added to all the wells and the plate was incubated again for 5–10 min in the darkroom at room temperature. The maximum optical absorption after half an hour was read using a spectrophotometer at a wavelength of 540 nm and the concentration of the samples was calculated using the standard curve. Based on the range of normal concentration of NOx as around 30 to 40 micromolar, the concentrations of 2, 12, 25, 50, 100, and 200 mmol/L were considered as the standard range.

### 2.3. Prostacyclin Measurement

A validated commercially available ELISA Kit for Prostacyclin measurement (Glory Science Co., Ltd. Company, Hemei, Taiwan, China) was used. First, the reagents were brought to room temperature. Fifty microliters of standard liquid were added to the first tube and 100 microliters were removed from the first tube and added to the second tube. Fifty microliters were taken from the second tube and added to the third tube and this process continued until the end. After removing the blank, 40 microliters of sample were added to the sample tubes and mixed. Then the solutions were incubated at 37 °C for 30 min. After incubation, washing solution was added and washed 5 times. Fifty microliters of HRP solution were added to each sample and incubated for 30 min for the second time. After the incubation, the washing step was performed again. In the next step, 50 uL of chromogen A and B were added to each tube except the blank, and the tubes were prevented from light exposure for 30 min. Then, 50 microliters of stopping solution was added and after 15 min, the optical absorption of the solutions was measured using a spectrophotometer at a wavelength of 450 nm. The measurement range was 50–150 ng/L.

### 2.4. Measurement of Thromboxane B_2_

A validated commercially available ELISA Kit for Human Thromboxane B_2_ measurement was used. First, the reagents were brought to room temperature. Fifty microliters of the standard liquid belonging to the kit itself was poured into each tube, then 100 microliters of the standard solution was added to the first tube, and 100 microliters was removed from the first tube and added to the second tube. Fifty microliters was taken from the second tube and added to the third tube and this process continued until the end. After removing the blank, 40 microliters of the sample was added to the sample tubes and mixed gently. Then, the solutions were incubated at 37 °C for 30 min. After incubation, the washing solution was diluted 30 times. In the next step, a washing solution was added to the samples and they were incubated for the second time for 30 min at the same temperature. After the incubation, the washing step was performed again. In the next step, 50 U of chromogen A and chromogen B were added to each tube except the blank, and the tubes were prevented from light exposure for 30 min. Then, 50 microliters of stopping solution was added and after 15 min, the optical absorption of the solutions was measured using a spectrophotometer at a wavelength of 450 nm. The measurement range was 1000–500 pg/mL.

### 2.5. Mediterranean Dietary Pattern Evaluation

The ten-item, ten-point Mediterranean Diet Adherence Screener (MEDAS) questionnaire was used to determine adherence to the MD. Based on the MEDAS ranking, participants in this study were divided into two groups: those who adhered to the MD less frequently (0–9) and those who adhered more frequently (10+). The frequency and percentage of responses to each item summed to total score calculation [39].

### 2.6. Statistical Analysis

The study sample size was calculated based on previous studies and by applying the following formula n = S_1_^2^ + S_2_^2^/(µ1 − µ2)^2^ f(α,β) = 36 × 36 + 45 × 45/(153 − 104)^2^ = 14.52 person for each group. Statistical analyses were performed with STATA Version 14.0 (College Station, TX, USA) for Windows. All continuous variables were reported as the mean± standard deviation. Independent T-test was used for assessment of the normal quantitative variables difference. The association between biochemical markers and adherence to the MD was also assessed using multivariate regression. In all analyses, a *p*-value *≤* 0.05 was considered as statistically significant.

## 3. Results

The general characteristics of the participants are shown in Table 1. The level of physical activity in healthy people was higher than angina patients (*p* = 0.02). Fifty-eight percent of Prinzmetal angina patients had a low level of physical activity, 38.2% had moderate activity, and 2.9% had vigorous activity. The Prinzmetal angina patients had significantly higher systolic and diastolic blood pressure than healthy subjects (*p* = 0.02).

The serum levels of Nitric oxide, Prostacyclin, and Thromboxane B_2_ in the participants are shown in Table 2. There was a significant difference in serum Nitric oxide, Thromboxane B_2_, and Prostacyclin between the two study groups. Nitric oxide level was 28.9 mmol/L in Prinzmetal angina patients and 34.8 in healthy subjects (*p* = 0.04). The Prostacyclin level mean in patients was significantly lower than healthy people (78.42 ± 10.97 vs. 85.59 ± 12.66 *p* = 0.01).

Table 3 indicates the results of the serum Nitric oxide, Prostacyclin, and Thromboxane B_2_ (as dependent variables) association with adherence to the Mediterranean diet. The serum Nitric oxide in patients with more adherence to MD was higher than in other patients (coeff. = 0.41 *p* = 0.04). The serum Prostacyclin level in patients with greater adherence was 0.34 units higher than patients with less adherence (coeff. = 0.34 *p* = 0.02) and this association was significant after adjusting for smoking, waist circumference, and physical activity level (coeff. = 0.22 *p* = 0.01). A negative association was observed between the level of serum Thromboxane B_2_ and adherence to the Mediterranean diet (coeff. = −0.48 *p* = 0.04). There was a significant negative association between systolic blood pressure and diastolic blood pressure with adherence to the Mediterranean diet (*p* ≤ 0.05).

The results related to the positive responses of the study participants to the intake of the Mediterranean diet components are reported in Table 4. The consumption of olive oil (13.13% vs. 48.33), vegetables (18.33% vs. 53.32%), fruits (23.33 vs. 70%), legumes (5% vs 65%), fish (5% vs. 66.66%), and nuts (3% vs. 48.33%) in Prinzmetal angina patients were significantly lower than in healthy individuals (for all, *p* < 0.05).

## 4. Discussion

Our study is the first study that has examined the level of adherence to the Mediterranean diet in Prinzmetal angina patients compared with healthy individuals, and the association of adherence to MD with serum levels of Nitric oxide, Prostacyclin, and Thromboxane B_2_. The results of the study show that the serum levels of Nitric oxide and Prostacyclin in Prinzmetal angina patients are significantly lower than in healthy individuals, and the patients’^,^ serum Thromboxane B_2_ is significantly higher than in healthy persons. Nguyen et al. indicated that the cause of spasms in these patients is the low production of Nitric oxide in the vascular endothelium [13]. Acetylcholine is released from parasympathetic nerves during rest [40]. Acetylcholine stimulates the endothelium to produce nitric oxide [41]. Endothelial damage leads to a lack of proper endothelial response to acetylcholine to produce Nitric oxide [42].

We also observed a significant association between adherence to the Mediterranean diet and serum Nitric oxide in Prinzmetal angina patients. More adherence to the Mediterranean diet led to an increase in the serum levels of Nitric oxide and Prostacyclin and a decrease in the serum level of Thromboxane B_2_. According to Shannon’s research, adherence to the Mediterranean diet boosts the production of Nitric oxide [43]. Nuts, legumes, unrefined grains, and seafood are abundant in the Mediterranean diet and are all major sources of L-arginine [43]. The Nitric oxide synthase enzymes oxidize this semi-essential amino acid to produce NO [44]. Li et al. [45] evaluated the potential efficacy of L-arginine supplementation in mitigating the adverse cardiovascular effects of hypertensive adults. In his study, there were no substantial changes in L-arginine-NO metabolites and inflammatory biomarkers. In the aforementioned study, physical activity was not measured, therefore, it can be one of the reasons for different results.

Some foods in the Mediterranean diet such as onion and garlic, fish, peas, beans, peanuts, soybeans, almonds, and walnuts are dietary sources of Citrulline. Citrulline is a precursor of Arginine and can play an indirect role in the production of Nitric oxide. Holguin et al. [46] indicated that short-term L-citrulline treatment improved the excretion of NO in obese asthmatics persons. Another study by Kim et al. [47] evaluated the age association of vascular dysfunction in older adults with the insufficient synthesis of Nitric oxide and showed that Citrulline ingestion improved impaired NO synthesis in older heart failure adults but not reactive hyperemic forearm blood flow (RH-FBF), suggesting that some factors other than NO synthesis play a role in the impaired RH-FBF in older heart failure adults, and/or it may require a longer duration of supplementation to be effective in improving RH-FBF.

Our results indicate that the consumption of olive oil in Prinzmetal angina patients was less than in healthy people. In the PREDIMED intervention study results [48], individuals who adhere more to the Mediterranean diet with extra virgin olive oil or nuts had a more plasmatic indicator of NO availability (sum of plasma nitrate and nitrite). Le Sayec et al. [49] indicated that olive oil phenolic and their circulating metabolites can modulate NO balance by decreasing its degradation.

In the present study, the amount of seafood consumption in Prinzmetal angina patients was lower than healthy people. Based on other studies, more intake of fresh fish is a protective factor against cardiovascular diseases [50,51], but some studies have shown inconsistent results. Krittanawong et al. [50], in a review study, concluded that non-fried fish consumption was probably associated with a reduced risk of overall cardiovascular disease events and myocardial infarction risk. In contrast, fried fish consumption is probably associated with an increased risk of overall cardiovascular disease events and myocardial infarction risk. Fish and sea foods contain multiple nutrients, such as omega-3 long-chain polyunsaturated fatty acids, protein, vitamin D, vitamin B, Calcium, Selenium, and other components. Several studies reported that omega-3 fatty acids could reduce cardiovascular disease risk via several mechanisms, including improving vascular function [52]. The omega-3 fatty acids precisely activate cardiovascular protective signaling pathways, increase the production of NO, and reduce oxidative stress and inflammation [53].

The fruit and vegetable consumption in the patients of this study was lower than in the healthy subjects. Ruxton et al. [54], in a review study, concluded that the consumption of fruit juice reduced the risk of stroke. Fruits and vegetables are rich in flavonoids [55]. Flavonoid-rich foods like apples, grapes, and red-orange juice, have shown positive effects on endothelial function [56]. Macready et al. [57] conducted one study to measure the dose-response relation between high-flavonoid (HF), low-flavonoid (LF), and habitual fruit & vegetables (F&Vs) intake and vascular function and other cardiovascular disease risk indicators. This study’s results indicated that HF F&Vs increased plasma NO (*p* = 0.0243) with 14 portions/day as a whole. McCall et al. [58], in another clinical study, showed a dose-dependent increase in endothelium-dependent microvascular function after consuming 1, 3, or 6 mixed F&V portions daily for 8 weeks in a parallel design. These results support this improvement in microvascular function from a total of 3.9 to 6.3 portions of F&Vs daily.

Chen et al. [59], in a randomized clinical trial, investigated the effect of Fruitow (FF), a water-soluble tomato extract, on platelet function in elderly persons and indicated that Fruitow blocked platelet aggregation and decreased Thromboxane B_2_ levels in elderly people. Consumption of Mediterranean diet foods reduced the thrombotic state (decrease in plasma von Willebrand factor, tissue factor pathway inhibitor, and tissue plasminogen activator inhibitor 1) [60]. A Mediterranean diet high in Oleic acid reduces the production of Thromboxane B_2_, decreases plasma von Willebrand factor (derived from the endothelium and is important in the process of coagulation during a platelet thrombus), and Plasminogen activator inhibitor 1 [60,61]. It can be concluded that adherence to the Mediterranean diet leads to improved endothelial function and prevents Prinzmetal angina-related spasms.

The use of retrospective dietary information is the limitation of the current study. It is suggested that future studies be conducted in the form of clinical intervention studies to confirm better the MD effect on Prinzmetal angina syndrome.

## 5. Conclusions

The serum level of Thromboxane B_2_ in Prinzmetal angina patients is higher than in healthy individuals. Prostacyclin and Nitric oxide serum levels in Prinzmetal angina patients are lower than in healthy people. More adherence to the Mediterranean diet led to a decrease in the serum Thromboxane B_2_ level and an increase in the serum Nitric oxide and Prostacyclin levels in patients with Prinzmetal angina. The amount of consumption of olive oil, fruits, vegetables, and legumes in healthy people was more than that of Prinzmetal angina patients.

## Figures and Tables

**Table 1:** General characteristics of study participants.

Variables	Prinzmetal Angina Patients *n* = 100	Healthy Controls *n* = 100	*p* *
Age (years)	51.26 ± 0.86	51.37 ± 0.97	0.651
Gender			
Male	50 [50%]	50 [50%]	0.79
Female	50 [50%]	50 [50%]	
Weight [kg]	78.17 ± 13.12	78.16 ± 8.81	0.81
Body mass index [kg/m^2^]	26.25 ± 1.12	26.15 ± 3.28	0.62
Waist circumference [cm]	95.35 ± 18.38	94.44 ± 12.04	0.58
Waist circumference to hip circumference	0.91 ± 0.14	0.82 ± 0.10	0.02 *
Physical activity [%]			0.02 *
- Low	58%	25%	
- Moderate	38.2%	44%	
- Vigorous	2.9%	21%	
Smoking habit [%]	48.5%	21.3%	0.01 *

* Student-*t* test for independent samples or chi-square test.

**Table 2:** Serum Nitric oxide, Prostacyclin, and Thromboxane B_2_, and blood pressure in study groups.

Variables	Prinzmetal Angina Patients	Healthy Controls	*p* *
Nitric oxide mmol/L	28.9 ±1.15	34.8 ± 6.25	0.04
Prostacyclin pg/mL	78.42 ± 10.97	85.59 ± 12.66	0.01
ThromboxaneB_2_ pg/mL	888.62 ± 43.58	553.21 ± 8.25	0.05
SBP mmHg	130.01 ± 28	118.6 ± 18	0.04
DBP mmHg	95.02 ± 9.35	70.92 ± 2.82	0.02

* Student-*t* test for independent samples.

**Table 3:** Association between adherence to the Mediterranean diet with serum levels of Thromboxane B_2_, Prostacyclin, and Nitric oxide in the study groups.

Dependent Variables		Prinzmetal Patients				Overall Healthy Subjects		
		coeff.f.	95%	*p* *		coeff.f.	95% CI	*p* *
NO	Model 1	0.41	0.38, 0.52	0.04	MODEL 1	0.31	0.28, 0.42	0.05
	Model 2	0.28	0.21, 0.31	0.02	MODEL 2	0.26	0.22, 0.34	0.01
PGI_2_	Model 1	0.34	0.29, 0.41	0.02	MODEL 1	0.51	0.48, 0.52	0.05
	Model 2	0.25	0.22, 0.29	0.01	MODEL 2	0.38	0.31, 0.41	0.04
TBb_2_	Model 1	−0.48	−0.42, −0.51	0.04	MODEL 1	−0.28	−0.21, −0.31	0.02
	Model 2	−0.23	−0.19, −0.28	0.02	MODEL 2	−0.22	−0.18, −0.25	0.04

Model 1: adjusted for age and gender; Model 2: Model 1 with further adjustments for smoking habit, waist size, and physical activity level. * Student-*t* test for independent samples.

**Table 4:** Positive answers to the MEDAS questionnaire.

	Prinzmetal Angina Patients [*n* = 60]		Healthy Persons [*n* = 60]		*p*
Olive oil, the main dressing	8	[13.13%]	29	[48.33%]	0.05
Olive oil, 4 ts/day	5	[8.33%]	28	[46.66%]	0.03 *
Vegetables, 2 s/day	11	[18.33%]	32	[53.33%]	0.01 *
Fruits, 3 s/day	14	[23.33%]	42	[70%]	0.04 *
Red meat, <1 s/day	5	[8.33%]	28	[46.66%]	0.04 *
Butter, <1 s/day	3	[5%]	29	[48.33%]	0.05
Sweet beverage, <1 s/day	3	[5%]	18	[30%]	0.02 *
Legumes, 3 s/week	5	[8.33%]	39	[65%]	0.01 *
Fish and seafood, 3 s/week	3	[5%]	40	[66.66%]	0.04 *
Sweets, <3 s/week	7	[11.66%]	20	[33.33%]	0.01 *
Nuts, 3/week	2	[3%]	29	[48.33%]	0.03 *
White meat over red	5	[8.33%]	28	[46.66%]	0.01 *

Notes: Data are expressed as numbers and percentages in parenthesis [*n*[%]] for categorical variables. Positive answers to the MEDAS questionnaire: Vegetables daily serving: 1 medium portion = 200 g; fruit daily serving: 1 serving = 100–150 g portion; red meat/hamburgers/other meat daily serving: 1 medium portion = 100–150 g; butter, margarine, or cream daily serving: 1 medium portion = 12 g; sweet or sugar-sweetened carbonated beverages daily serving: 1 medium portion = 200 mL; legumes weekly serving: 1 portion = 150 g; fish daily serving: 1 medium portion = 100–150 g; seafood daily serving: 1 medium portion = 200 g; nuts weekly serving: 1 portion of dairy product = 30 g. MEDAS: Mediterranean diet adherence screener; MD: Mediterranean diet; s: serving; ts: tablespoon; *: *p* < 0.05.

## Data Availability

Data are unavailable due to local privacy restrictions.
